# Officers American Medical Association

**Published:** 1882-07-20

**Authors:** 


### *OFFICERS OF AMERICAN MEDICAL ASSOCIATION.*

### REPORT OF THE COMMITTEE ON NOMINATIONS.

President, Dr. John L. Atlee, Lancaster, Pa.; First Vice-President, Dr. Eugene Grissom, Raleigh, N. C.; Second Vice-President, Dr. A. J. Stone, St. Paul, Minn.; Third Vice-President, Dr. J. A. Octerlony Louisville, Ky.; Fourth Vice-President, Dr. H. S. Orme, Los Angelos, Cal.; Treasurer, Dr. R. J. Dunglison, Philadelphia, Pa.; Librarian, C. H. A. Kleinschmidt, Washington, D. C.

Judicial Council to fill vacancies—Dr. N. S. Davis, Ill.; Dr. J. M. Brown, U. S. N.; Dr. X. C. Scott, O.; Dr. M. Sexton, Ind.; Dr. N. C. Husted, N. Y.; Dr. Wm. Lee, Md.; Dr. J. E Reeves, West Virginia.

The next place of meeting—Cleveland, O.

Chairman of Committee of Arrangements—Dr. X. C. Scott, Cleveland, O.

Section in Practice of Medicine—J. H. Hollister, of Chicago, Chairman; John G. Lee, Philadelphia, Secretary.

Section in Surgery and Anatomy—W. F. Peck, Davenport, Iowa, Chairman; Paul F. Eve, Nashville, Tenn., Secretary.

Section in Obstetrics—John K. Bartlett, Milwaukee, Wis., Chairman; G. A. Moses, St. Louis, Mo., Secretary.

Section in Medical Jurisprudence and State Medicine—Foster Pratt, Kalamazoo, Mich., Chairman; Thos. L. Neal, Dayton, O., Secretary.

Section in Ophthalmology, Otology and Laryngology—A. W. Calhoun, Atlanta, Ga., Chairman; Carl Seiler, Philadelphia, Pa., Secretary.

Section in Diseases of Children—R. F. Blount, Wabash, Ind., Chairman; J. H. Sears, Texas, Secretray.

Section in Dentistry—D. H. Goodwillie, N. Y., Chairman; T. W. Brophy, Chicago, Ill., Secretary.

Committee on Necrology—J. M. Toner, D. C., Chairman.
Committee on Publication—W. B. Atkinson, Chairman.
Assistant Secretary—Dr. I. N. Hines, Cleveland, O.
Committee on State Medicine—D. C. Ewing, Ark., Chairman.

---

DR. J. E. JANVRIN, of New York, requests us to call the attention of our readers to the fact that a "System of Gynæcology by American Authors" is in process of preparation, and it is intended that this shall be as nearly encyclopædic as possible, and that to him has been allotted the task of writing the chapter on the "History and Statistics of Ovariotomy," and he asks information from medical brethren on this subject. It is desirable, too, to have all material ready for the press by the end of this year—so that the sooner the returns are made, the lighter will be the task of the editor. Please request all who wish their cases published, to send me the reports before September 1, prox.

May I request your earnest co-operation in the matter, that the work may be worthy the great subject in hand. The questions to be answered are as follows:

1. Name of Operator?
2. Age of Patient?
3. Nationality?
4. Married or single?
5. Aspiration or previous tapping?
6. Duration of Growth?
7. Laparotomy or vaginal Operation?
8. Condition of patient at time of Operation?
9. Were antiseptic precautions used?
10. Was the spray used?
11. Long or short incision?
12. Adhesions or other complications?
13. Double or single Ovariotomy?
14. Pathological features of Cyst?
15. Treatment of the Pedicle?
16. With or without Drainage?
17. Duration of Operation?
18. Complicated or Uncomplicated history of Operation?
19. Anti-Pyretics used, if any?
20. Result. Cause of death, if any?
21. Primary or secondary operation?

Let the answers be as concise as possible. In many cases a simple yes or no will suffice.

All communications should be addressed to him, 191 Madison Avenue, New York City.

